# Line-field confocal versus conventional optical coherence tomography: A head-to-head comparison in basal cell carcinoma diagnosis

**DOI:** 10.1016/j.jdin.2026.04.007

**Published:** 2026-04-16

**Authors:** Tom Wolswijk, Patricia Joan Nelemans, Janne de Kort, Lisa Hillen, Klara Mosterd

**Affiliations:** a Department of Dermatology, Maastricht University Medical Center+, Maastricht, the Netherlands; b GROW Research Institute for Oncology and Reproduction, Maastricht University, Maastricht, the Netherlands; c Department of Epidemiology, Maastricht University, Maastricht, the Netherlands; d Faculty of Health Medicine and Life Sciences, Maastricht University, Maastricht, the Netherlands; e Department of Pathology, Maastricht University Medical Center+, Maastricht, the Netherlands

**Keywords:** basal cell carcinoma, BCC, line-field confocal optical coherence tomography, LC-OCT, OCT, optical coherence tomography

*To the Editor:* Noninvasive imaging techniques, such as conventional optical coherence tomography (cOCT) and line-field confocal optical coherence tomography (LC-OCT), provide a noninvasive alternative to invasive biopsy for diagnosing and subtyping basal cell carcinoma (BCC), thus allowing for immediate treatment without awaiting histopathological results (Supplement I, available via https://data.mendeley.com/datasets/chkdfhbzmx/1).^1^^,^^2^ LC-OCT provides better resolution but less signal depth, possibly improving BCC detection while risking missing deeper lesions. This diagnostic cohort study aimed to cf the diagnostic accuracy for BCC detection and subtyping between LC-OCT and cOCT.

In this head-to-head comparison, clinically equivocal lesions suspect for BCC with an indication for biopsy were scanned with cOCT and LC-OCT (Supplement II, available via https://data.mendeley.com/datasets/chkdfhbNON-zmx/1). An OCT assessor, experienced with both devices evaluated scans for BCC presence and subtype in a random nonpaired order alongside clinical and dermoscopic photographs. Diagnostic confidence was graded on a 5-point scale (Table I). Two analyses were performed: (1) detection of BCC presence (score ≥3), and (2) detection of BCC with certainty on subtype (score 4). For subtype discrimination, sensitivity was defined as the proportion of a specific BCC subtype (ie, superficial (sBCC), nodular (nBCC), or infiltrative (iBCC), that was correctly classified as such by the assessor. Specificity was defined as the proportion of the other two subtypes correctly classified as such (ie, sBCC/nBCC, sBCC/iBCC or nBCC/iBCC). Histopathology served as the reference standard. In case of BCC, the most aggressive subtype was considered the true subtype for both histopathologic and OCT assessment (sBCC, least aggressive and iBCC, most aggressive), because clinical decisions are guided by the most aggressive tumor features. The assessor was blinded to histopathology (TW), whereas the dermatopathologist was blinded to OCT scans (LH).

**Table I:** Five-point confidence scale for diagnosing basal cell carcinoma on optical coherence tomography

Confidence score	Definition
4	Certain of BCC presence and its subtype. Biopsy could be omitted
3	Certain of BCC presence, uncertain of subtype. Biopsy needed for subtyping
2	High suspicion for BCC
1	Low suspicion for BCC
0	BCC is not in differential diagnosis

*BCC*, Basal cell carcinoma.

A total of 139 patients with 197 lesions were included with a BCC prevalence of 58.9% (Supplement II, available via https://data.mendeley.com/datasets/chkdfhbzmx/1). A high-confidence BCC diagnosis (score 4) was established in 102 lesions (51.8%) on LC-OCT and in 86 lesions (43.7%) on cOCT. LC-OCT showed higher sensitivity than cOCT for detecting BCC with subtype certainty (85.3% vs 70.7%, *P* = .003), at similar specificity (96.3% vs 95.1%, *P* > .999). Positive predictive values were 97.1% for LC-OCT and 95.3% for cOCT (*P* = .564), and negative predictive values were 82.1% and 69.4%, respectively (*P* = .036). When subtype certainty was not required, the difference in sensitivity was smaller (86.2% vs 82.8%, *P* = .523). Sensitivity for detecting infiltrative subtypes was lower for LC-OCT (48.0% vs 77.8%, *P* = .049), whereas sensitivity for sBCC and nBCC detection was comparable between both modalities (Table II).

**Table II:** Diagnostic parameters for BCC detection and subtyping on line-field confocal optical coherence tomography and conventional optical coherence tomography depending on cut-off point for a positive OCT test result

	LC-OCT % (x/*n*)	95% CI	cOCT % (x/*n*)	95% CI	*P* value
Certain of BCC presence and subtype (4 vs 3210)					
Sensitivity	85.3 (99/116)	81.1-87.2	70.7 (82/116)	66.0-73.0	.003
Specificity	96.3 (78/81)	90.2-99.0	95.1 (77/81)	88.4-98.4	>.999
PPV	97.1 (99/102)	92.2-99.2	95.3 (82/86)	89.0-98.5	.564
NPV	82.1 (78/95)	76.9-84.4	69.4 (77/111)	64.5-71.8	.036
Only certain of BCC presence (43 vs 210)					
Sensitivity	86.2 (100/116)	82.0-88.1	82.8 (96/116)	77.9-85.9	.523
Specificity	96.3 (78/81)	90.3 - 99.0	91.4 (74/81)	84.4-95.9	.219
PPV	97.1 (100/103)	92.3-99.2	93.2 (96/103)	87.7-96.8	.195
NPV	83.0 (78/94)	77.8-85.3	78.7 (74/94)	72.7-82.6	.458

sBCC	LC-OCT % (x/*n*)	95%CI	cOCT % (x/*n*)	95% CI	*P* value
Sensitivity (sBCC)	81.0 (17/21)	62.7-91.8	84.2 (16/19)	65.3-94.3	.807
Specificity (non-sBCC)	94.9 (74/78)	90.0-97.8	95.2 (60/63)	86.5-98.3	.935
PPV	81.0 (17/21)	62.7-91.8	84.2 (16/19)	65.3-94.3	.807
NPV	94.9 (74/78)	90.0-97.8	95.2 (60/63)	89.5-98.3	.935

nBCC	LC-OCT % (x/*n*)	95% CI	cOCT % (x/*n*)	95% CI	*P* value
Sensitivity (nBCC)	79.2 (42/53)	69.6-86.9	73.3 (33/45)	62.9-80.9	.503
Specificity (non-nBCC)	71.7 (33/46)	60.6-80.5	81.1 (30/37)	68.4-90.3	.323
PPV	76.4 (42/55)	67.1-83.7	82.5 (33/40)	70.8-91.0	.469
NPV	75.0 (33/44)	63.4-84.2	71.4 (30/42)	60.3-79.6	.708

iBCC	LC-OCT % (x/*n*)	95%CI	cOCT % (x/*n*)	95% CI	*P* value
Sensitivity (iBCC)	48.0 (12/25)	30.9-63.7	77.8 (14/18)	55.8-91.9	.049
Specificity (non-iBCC)	85.1 (63/74)	79.4-90.4	85.9 (55/64)	79.8-89.9	.894
PPV	52.2 (12/23)	33.6-69.2	60.9 (14/23)	43.7-71.9	.552
NPV	82.9 (63/76)	77.3-88.0	93.2 (55/59)	86.5-97.5	.073

*BCC*, Basal cell carcinoma; *iBCC*, infiltrative basal cell carcinoma; *LC-OCT*, line-field confocal optical coherence tomography; *nBCC*, nodular basal cell carcinoma; *NPV*, negative predictive value; c*OCT*, conventional optical coherence tomography; *PPV*, positive predictive value; *sBCC*, superficial basal cell carcinoma.

The difference in diagnostic accuracy between both modalities may vary between assessors with differing levels of experience per modality and larger studies with multiple OCT assessors are needed to validate the findings in this study.

In conclusion, the difference in sensitivity depends on whether BCC subtype certainty is required. Without this requirement, performance is comparable between devices. However, when subtype certainty is required, LC-OCT yields more high-confidence diagnoses, and higher sensitivity without compromising specificity. Although this potentially allows for more biopsies to be omitted, this advantage must be weighed against the risk of missing deeper growing iBCCs.

### Declaration of Generative AI and AI-Assisted Technologies in the Writing Process

During the preparation of this work the authors used DeepL Write and ChatGPT in order to optimize the readability of the text. After using this tool/service, the authors reviewed and edited the content as needed and take full responsibility for the content of the publication.

## Conflicts of interest

None disclosed.
